# Focused cardiac ultrasound in resuscitation

**DOI:** 10.1186/cc12237

**Published:** 2013-03-19

**Authors:** E Fish, L Fuchs, G Gulati, D Talmor, A Oren-Grinberg

**Affiliations:** 1 Beth Israel Deaconess Medical Center, Boston, MA, USA

## Introduction

Focused cardiac ultrasound is recognized as a vital tool in critical care medicine. Few studies, however, have examined the utility of this modality in resuscitation. While integration of ultrasound can supplement resuscitation by potentially establishing the etiology of cardiac arrest, it must be done safely to avoid interruption of compressions. The aim of this study was to examine the integration of focused cardiac ultrasound into resuscitations in our hospital.

## Methods

We performed a retrospective observational study of patients undergoing resuscitation at Beth Israel Deaconess Medical Center, Boston, USA, from 2009 to 2012. Inclusion criteria were age >17 years and performance of focused cardiac ultrasound during resuscitation. Recorded variables included admission data, code demographics and diagnosis, therapy outside standard ALS protocol, time to return of spontaneous circulation (ROSC), and outcomes data.

## Results

**Figure 1:**
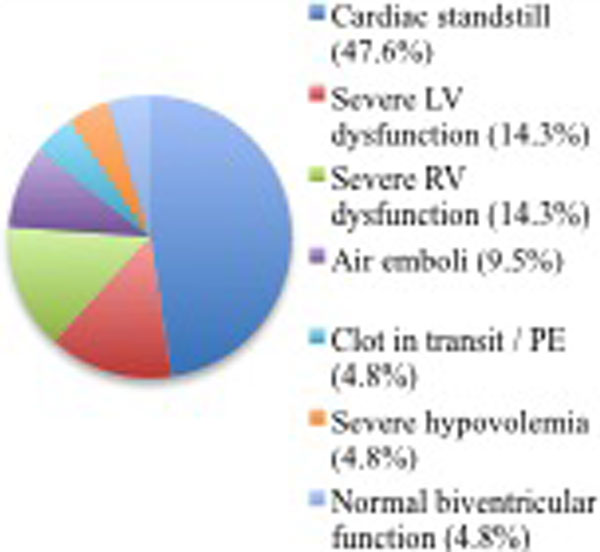
**Ultrasound findings**.

Of 33 eligible patients, 12 were excluded due to incomplete cardiac ultrasound reports; 21 patients were enrolled. Cohort demographics included: 57.1% male, average age 64.3 years, average BMI 28.5 kg/m^2^, average Charlson score 5.5. Resuscitations took place on the wards (52.4%), ICU (42.9%), or operating room (4.8%). Most patients had an initial unshockable rhythm (71.5%). The most common ultrasound finding was cardiac standstill (47.6%) (Figure 1). The most common intervention as a result of the ultrasound was initiation of a pressor infusion (33.3%), of which 71.4% were ionotropes. Additional therapies included blood transfusion (4.8%), heparin (9.5%), tPA (4.8%), cardiac catheterization (4.8%), and surgery (9.5%). ROSC was achieved in 37.5% of patients; average time to ROSC was 13 minutes. A total 33.3% of patients who underwent ALS were alive at hospital discharge and 28.6% at 1 year.

## Conclusion

Focused cardiac ultrasound is a feasible adjunct to ALS resuscitation and may assist in the early identification of reversible causes of cardiac arrest. Care must be taken to ensure no interruptions to cardiac compressions are made by performance during pulse checks. Further studies are needed to examine the outcomes associated with its integration into resuscitations.
